# All-optical switching in epsilon-near-zero asymmetric directional coupler

**DOI:** 10.1038/s41598-022-22573-7

**Published:** 2022-10-26

**Authors:** Yanhua Sha, Ze Tao Xie, Jiaye Wu, H. Y. Fu, Qian Li

**Affiliations:** 1 School of Electronic and Computer Engineering, Peking University, Shenzhen, 518055 China; 2 École Polytechnique Fédérale de Lausanne (EPFL), STI-IEM, 1015 Lausanne, Switzerland; 3 Tsinghua Shenzhen International Graduate School, Tsinghua University, Shenzhen, 518055 China

**Keywords:** Integrated optics, Optics and photonics

## Abstract

We propose an all-optical switch based on an asymmetric directional coupler structure with epsilon-near-zero (ENZ) layer. The nonlinear optical properties the of ENZ layer are analyzed by hot-electron dynamics process, and the all-optical operating performance of the switch on the silicon nitride platform is investigated. It is found that the pump-induced refractive index change in ENZ layer gives rise to a transfer of signal light in the optical system. We demonstrate that the proposed switch design features an insertion loss of < 2.7 dB, low crosstalk of < − 18.93 dB, and sub-pico-second response time at the communication wavelength of 1.55 μm. With ultrafast response, high performance, and simple structure, the device provides new possibilities for all-optical communication and signal processing.

## Introduction

Exploration of on-chip optical computing and signal processing has emerged in chip-scale all-optical compute-and-store system^1^ and advanced all-optical neurosynaptic network^2^. As fundamental elements, optical logic gates play an important role in complex integrated photonic systems and achieve significant improvements in speed and power consumption of very large-scale integrated photonics. In conventional silicon-based switches, the control mechanism is mainly refractive index change due to two-photon absorption-induced optical Kerr nonlinearity^3^. However, such devices suffer from high-power consumption and large footprint. These drawbacks can be overcome by designed resonance structures, namely, micro-ring resonators^4^, photonic crystals^5^, etc. Yet, resonance structures are inevitably accompanied by the narrow optical bandwidth and the high demand for fabrication tolerance. To mitigate the disadvantages of silicon photonics, a variety of novel materials integrated with silicon platforms have been demonstrated, such as two-dimensional materials (graphene^6^, black phosphorus^7^, transition metal dichalcogenides^8^) and phase-change materials (Ge_2_Sb_2_Te_5_^9^ and VO_2_^10^).

In the recent two decades, transparent conductive oxide (TCO) such as indium tin oxide (ITO)^11^, aluminum-doped zinc oxide (AZO)^12^, cadmium oxide (CdO)^13^ have attracted considerable interest in epsilon-near-zero (ENZ) spectral region. Such media exhibit a vanishing permittivity at ENZ wavelength, giving rise to a series of new physical phenomena^14^, such as enhanced electric field^15^, large optical nonlinearity^11^, ultrashort pulse interaction^16^, etc., and advanced applications including electro-optical modulator^17,18^, all-optical switch^19^, perfect absorber^13^, etc. For all-optical switching, ENZ TCO materials also have several advantages, such as greater refractive index change by pump excitation, ultrafast response time^11^, and intraband optical excitation^13^. These characteristics of ENZ TCO can provide possibilities for breaking the limitations of traditional all-optical waveguide logic gates. To date, all-optical ENZ switches are mostly based on metasurfaces^20,21^ or bulk^22^ structures, and the reported ENZ-related all-optical waveguide devices are mainly phase modulation designs^23,24^. Therefore, it is necessary to explore its form as all-optical switch in integrated photonic systems.

Here, we propose an all-optical switching scheme of an asymmetric directional coupler (ADC) structure, in which high mobility TCO material (dysprosium-doped CdO) and a pump excitation path are opted to achieve ultrafast and efficient all-optical switching. We analyze the thermal excitation and transient response of the ENZ TCO by employing the non-parabolic conduction band model and the two temperature model (TTM). The two-waveguide optical system consists of an input waveguide and a TCO-loaded waveguide, and the signal light is launched into the input waveguide. The ENZ TCO layer remains low index and lossy without pump irradiation, thus the TCO-loaded waveguide has minimal optical field confinement, resulting in a tendency for light to stay and propagate in the input waveguide. When the pump is irradiated to the ENZ TCO layer at 40 degrees off-normal, the TCO layer is triggered to have a high refractive index and a low absorption coefficient. Therefore, the TCO-loaded waveguide has large optical confinement to accomplish the coupling and transmission. This dedicatedly designed all-optical device based on the silicon nitride waveguide platform yields performance of low insertion loss (IL), low crosstalk (CT), broad bandwidth, as well as ultrafast optical response.

## Results and discussions

### Working principle of all-optical switching in epsilon-near-zero asymmetric directional coupler

**Figure 1:**
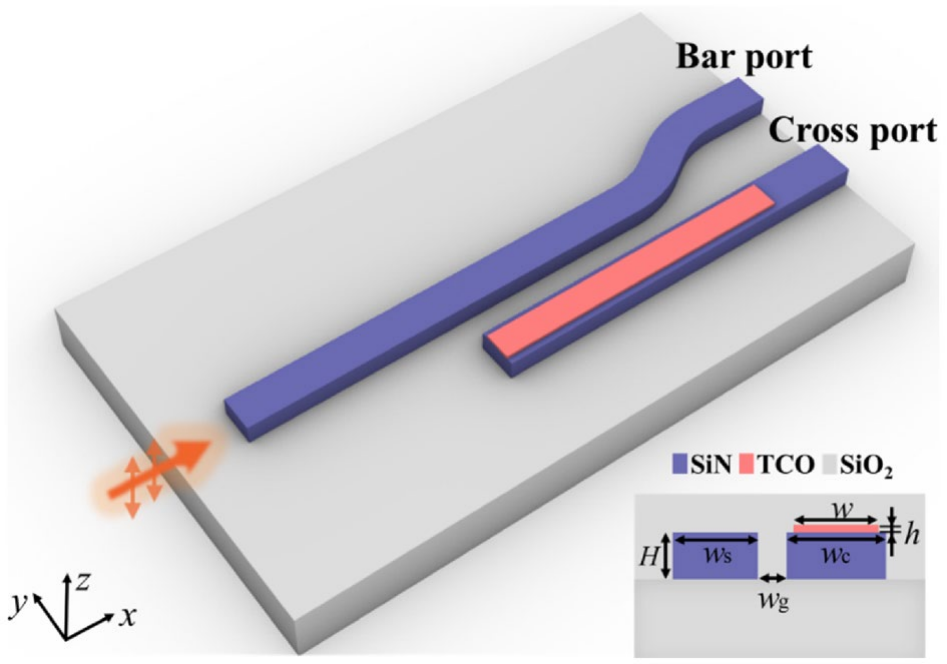
Perspective and cross-sectional view of the designed all-optical switch system. The proposed design includes an input waveguide and an ENZ TCO-loaded waveguide. The bar port and the cross port are outputs of the input waveguide and the TCO-loaded waveguide, respectively.

Figure 1 shows the perspective view and cross-sectional view of the proposed switch based on ADC. The switch system consists of two adjacent, parallel silicon nitride (SiN) waveguides: an input waveguide and a TCO-loaded waveguide. Geometric parameters are marked in the cross-sectional view, in which *H* is the height of the two waveguides, *h* and *w* are the height and width of TCO layer, *w*_g_ is the distance between the two waveguides, *w*_s_ and *w*_c_ are the width of the input waveguide and the TCO-loaded waveguide, respectively. The ENZ TCO features large refractive index change, electric field enhancement, and ultrafast nonlinear response under excitation of pump light, such optical properties can be applied to the directional coupler by elaborate design. In the scheme of the proposed all-optical switch, an out-of-plane TM-polarized pump pulse is employed to induce a change of refractive index in TCO layer, resulting in coupled and uncoupled states in the two-waveguide optical system. Under the static state, the difference in the effective index between the input waveguide and the TCO-loaded waveguide causes a phase mismatch due to the low index of TCO layer. Therefore, the launched guided mode is confined and propagates in the input waveguide. While TCO is excited to a larger refractive index by pump fluence, the effective indices of the two waveguides are identical and satisfy the condition of phase match. The launched guided mode in the input waveguide tends to couple to the TCO-loaded waveguide according to coupled-mode theory. Therefore, the structural parameters of the ADC are optimally chosen to ensure phase match in the coupled state and phase mismatch in the uncoupled state.

### Optical nonlinearity of ENZ layer

In the near-infrared region, degenerately doped semiconductors TCOs exhibit metal-like properties. In general, the following Drude model can quantify its permittivity dispersion profile^13^:

1 $$\varepsilon (\omega ) = \varepsilon_{\infty } - \frac{{\omega_{{\text{p}}}^{2} }}{{\omega^{2} + i\gamma \omega }},$$

where *ε*_∞_ is high-frequency permittivity, *γ* is the damping frequency, *ω* is the angular frequency of light and *ω*_p_ is plasma frequency. Here, we describe the static optical response of TCO with values from the references^23,25^, where *ε*_∞_ = 5.5, *γ* = 3.35 × 10^13^ rad s^−1^, *ω*_p_ = 2.85 × 10^15^ rad s^−1^. The real part of the permittivity has a zero-crossing at the wavelength of 1.55 μm.

**Figure 2:**
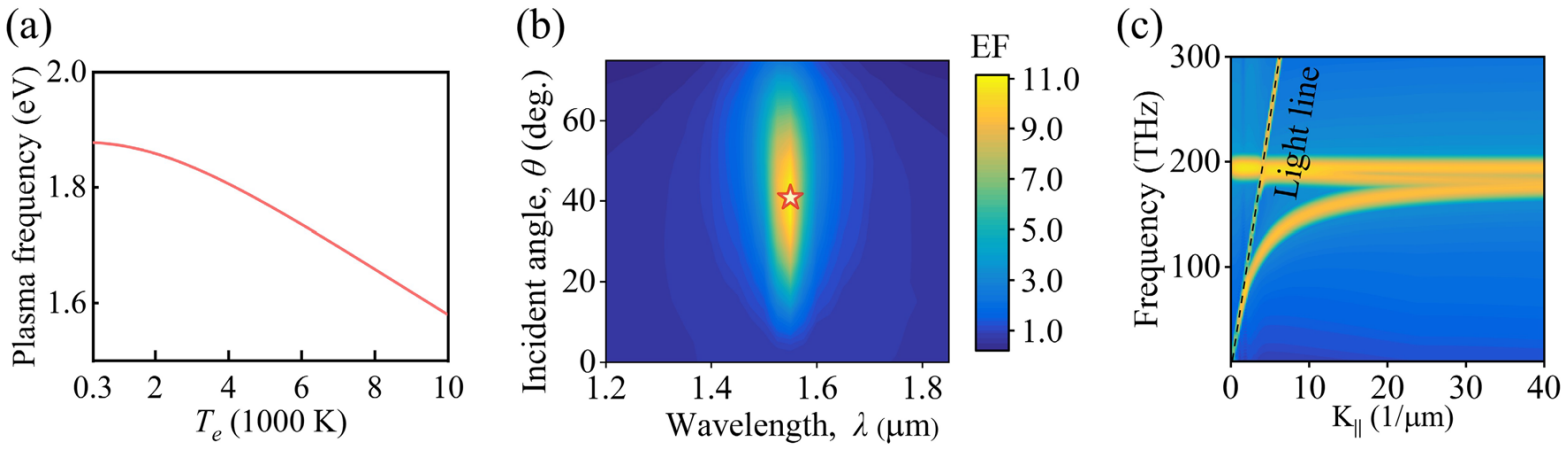
(**a**) Calculated plasma frequency *ω*_p_ as a function of hot-electron temperature *T*_e_. (**b**) Simulated enhancement factor as a function of wavelength and incident angle. The red star symbol indicates a maximum value. (**c**) Dispersion relation of the TCO layer.

The nonlinearity of TCO can be ascribable to modification in the energy distribution in its non-parabolic conduction band induced by pump, which can be written as (*ℏ*^2^*k*^2^/2* m*) = *E* + *CE*^2^^26,27^. After considering the non-parabolicity of the conduction band, the relation between the plasma frequency *ω*_p_ and the hot-electron temperature *T*_*e*_ of TCO can be calculated by Eq. (2) (see “Methods”: “Non-parabolic conduction of TCO” section for details). The calculated temperature-dependent plasma frequency is shown in Fig. 2a, and the plasma frequency *ω*_p_ decreases with the increment of the hot-electron temperature *T*_e_.

As intensively studied in the past, ENZ materials possess a pronounced angular-dependent nonlinearity response, which is associated with field enhancement occurring within the near-zero permittivity region^28^. Here, we calculate the field enhancement factor (EF =|*E*_TCO_|^2^/|*E*_0_|^2^, where *E*_0_ is the incident electric field in the air and *E*_TCO_ is the electric field inside the TCO ENZ layer) as a function of wavelength and incident angle^11^. The pronounced angular-dependent nonlinearity of TCO is observed in ENZ region. For a *p*-polarized incident light, the enhancement of the electric field within the TCO film enables larger nonlinearity^11,13, 28, 29^. This angular dependence can be calculated by considering the wavelength- and angle-dependent field enhancement factor EF^11,13, 29^. As shown in Fig. 2b, the field enhancement factor is up to 11.2-fold at an angle of 40° at ENZ wavelength, which is the largest nonlinearity that can be obtained in the ENZ TCO layer. Figure 2c further illustrates the dispersion relation of the TCO layer calculated by the finite-difference time-domain technique (Lumercial FDTD Solution). The calculation of bandstructure based on FDTD method is widely recognized and its accuracy has been verified^30–32^. In the FDTD bandstructure simulation of this work, a cloud of dipoles with different phases and orientations are placed inside the ENZ TCO film surrounded by a simulation region with Bloch boundary conditions. And the simulation time is set to be sufficiently long to ensure the appearance of modes that are supported by the ENZ TCO film. One can observe a nearly flat dispersion near 193.8 THz frequency, which is consistent with the maximum field EF at 1.55 μm (~ 193.4 THz). On the outside (right-hand side) of the light line cone, the thin ENZ film supports a long-range surface plasmon-polariton mode, which is known as ENZ mode for a thin ENZ film^30, 33–36^. A leaky mode emerges on the inside (left-hand side) of the light line cone. The excitation of this leaky mode is related to the Berreman absorption for a thin film, thus it is known as the Berreman mode^33–35^ or the Ferrell–Berreman mode^37^. The ENZ mode is a non-radiative mode which needs to be excited by coupling to localized plasmon resonances near the vertical component of electric field in ultrathin films of thickness < ≈ *λ*_ENZ_/50 with an additional coupling mechanism^34,36^. While the Ferrell–Berreman mode or Berreman mode is a radiative mode, it can be excited directly from the free space by a *p*-polarized light^34,36,38^. The large nonlinearity of ENZ media is associated with the enhancement of the electric filed^28^. While its enhancement mechanism originates from the continuity of the electric field, i.e., Maxwell’s boundary conditions^11,28^. For the ENZ TCO film, a *p*-polarized light at oblique incidence from free space leads to the excitation of the Ferrell–Berreman mode at the ENZ wavelength of 1.55 μm, resulting in an electric field enhancement^13,33^. This eventually allows the existence of large nonlinearity of ENZ TCO under proper pump irradiation.

**Figure 3:**
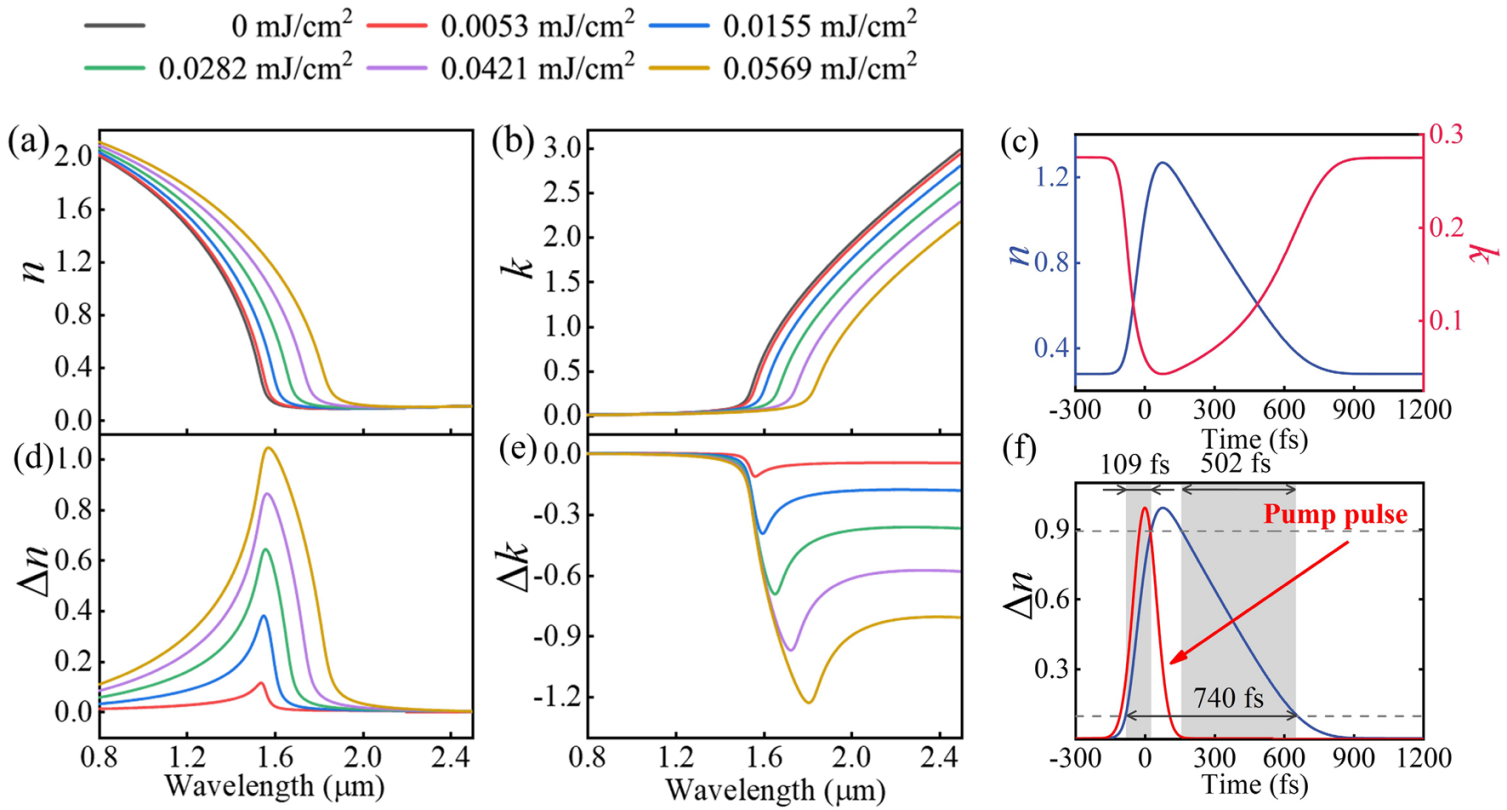
(**a**) Refractive index *n*, (**b**) absorption coefficient *k*, and (**c**) the transient response of *n* and *k* under *F* = 0.0569 mJ cm^−2^ at ENZ wavelength 1.55 μm. (**d**) The changes of refractive index Δ*n*, (**e**) absorption coefficient Δ*k*, and (**f**) the ultrafast change Δ*n* in time domain at ENZ wavelength. The red curve denotes the pulse intensity profile (in arbitrary units).

Furthermore, we utilize TTM (see “Methods” Two-temperature-model for details) to describe the temporal optical response of TCO. Based on TTM, change of dynamic hot-electron temperature *T*_*e*_ under pump fluence *F* can be derived, which can be later substituted into the non-parabolic conduction model to obtain the plasma frequency *ω*_p_ under different *F*. An increase in hot-electron temperature *T*_*e*_ will lead to a decreased *ω*_p_ and result in the modulation of permittivity of TCO. Consequently, refractive index *n*, absorption coefficient *k*, and their corresponding changes Δ*n*, Δ*k* can be obtained under different *F* based on the aforementioned results. Figure 3a,b show the refractive index *n* and absorption coefficient *k* of the ENZ TCO layer under various pump fluence *F*, while Fig. 3d,e further depict the changes of refractive index Δ*n* and absorption coefficient Δ*k.* One can observe both *n* and *k* have a larger modulation in the vicinity of ENZ spectral region. More specifically, the peak of Δ*n* occurs at ~ 1.55 μm, manifesting the enhanced nonlinearity response induced by ENZ effect in the TCO layer. For the scheme of the all-optical ADC switch, the TCO layer exhibits a sufficient refractive index change Δ*n* and a relatively low absorption coefficient *k* under *F* = 0.0569 mJ cm^−2^, which allows coupling between the input waveguide and the TCO-loaded waveguide. Therefore, we opt for a pump fluence of 0.0569 mJ cm^−2^ in the following device design. The temporal response of refractive index *n* and absorption coefficient *k* at ENZ wavelength under *F* = 0.0569 mJ cm^−2^ shown in Fig. 3c reveals the ultrafast optical properties of the TCO layer. Figure 3f further illustrates the transient Δ*n* response of the TCO layer. One can observe that the TCO layer has a rise time of ~ 109 fs and a recovery time of ~ 502 fs, corresponding to a high all-optical controlling speed of 1.35 THz in switching. To summarize, the ENZ TCO layer features a large refractive index change, low loss, and ultrafast transient response at ENZ wavelength.

### Scheme design of the optical switch

**Figure 4:**
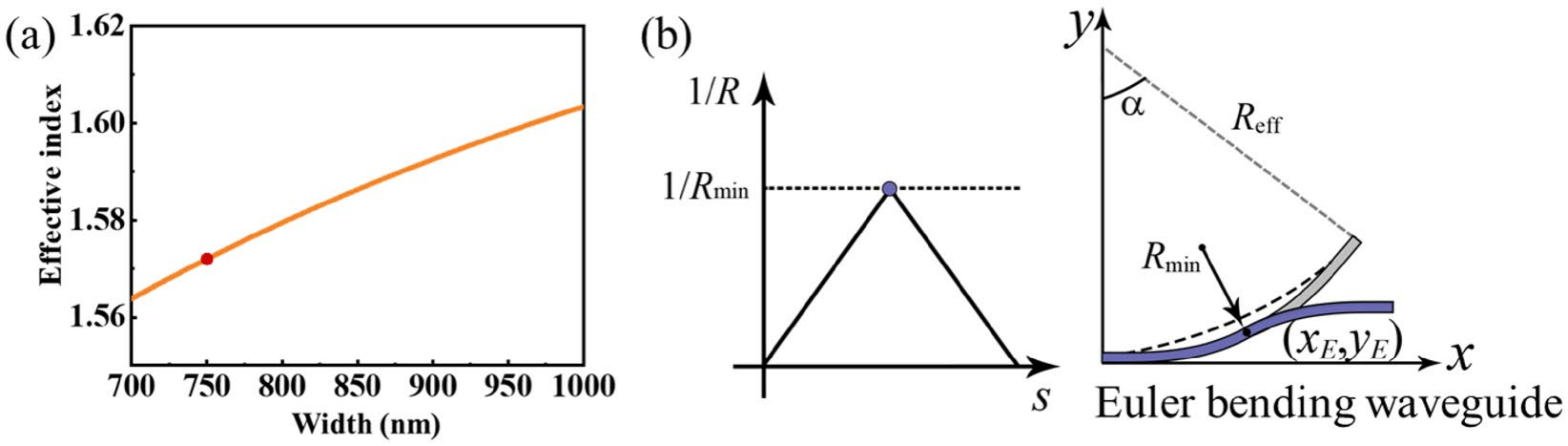
(**a**) Effective index of the TM mode as a function of width of the input waveguide *w*_s_, the phase-match point marked with a red dot. (**b**) The basic concept of the Euler bending waveguide used in the configuration.

Here, the two SiN waveguides are engineered to have identical height *H* = 450 nm, and *w*_*c*_ is set to 850 nm to ensure single-mode operation. The 60 nm-thick tunable TCO layer, which serves as a key component in the signal routing system is deposited on top of the waveguide. Considering the alignment precision of the electron-beam lithography, the width of TCO layer is chosen to be 100 nm narrower than the *w*_*c*_^39,40^. The supportable guided modes in the TCO-loaded waveguide and the input waveguide can be calculated by mode solver based on the finite element method (Lumerical Mode Solution). The results indicate that the effective index of the TCO-loaded waveguide under the pump-off state *F* = 0 and the pump-on state *F* = 0.0569 mJ cm^−2^ are 1.498 + 0.012*i* and 1.575 + 0.003*i*, respectively. The effective index of the input waveguide for various width *w*_*s*_ is shown in Fig. 4a. To ensure phase match between the TCO-loaded waveguide and the input waveguide under pump-on state, the input waveguide has to possess the same effective index as the TCO-loaded waveguide, thus *w*_*s*_ is set as 750 nm. At the end of the input waveguide, a designed Euler bending waveguide is utilized to make the two waveguides uncoupled. The Euler waveguide is a kind of bending waveguide designed on the basis of the mathematical Euler spiral, in which the curvature grows linearly with the curve length *s*, as shown in Fig. 4b. The curve length *s* is determined by 2*R*_min_*α*, where *R*_min_ denotes a given minimum curvature radius at the endpoint of (*x*_*E*_,*y*_*E*_) and *α* denotes a given angle. The whole Euler bending waveguide with length *s* consists of mirror-symmetric sections, each of them bends the light path by *α*/2^41^. The use of the Euler curve contributes to the reduced bending loss and radiative loss when light is bent from straight to bending waveguide^42^. In our work, the designed bending part of the Euler curve waveguide is set as *R*_min_ = 20 μm and *α* = 20°.

### Mode analysis

**Figure 5:**
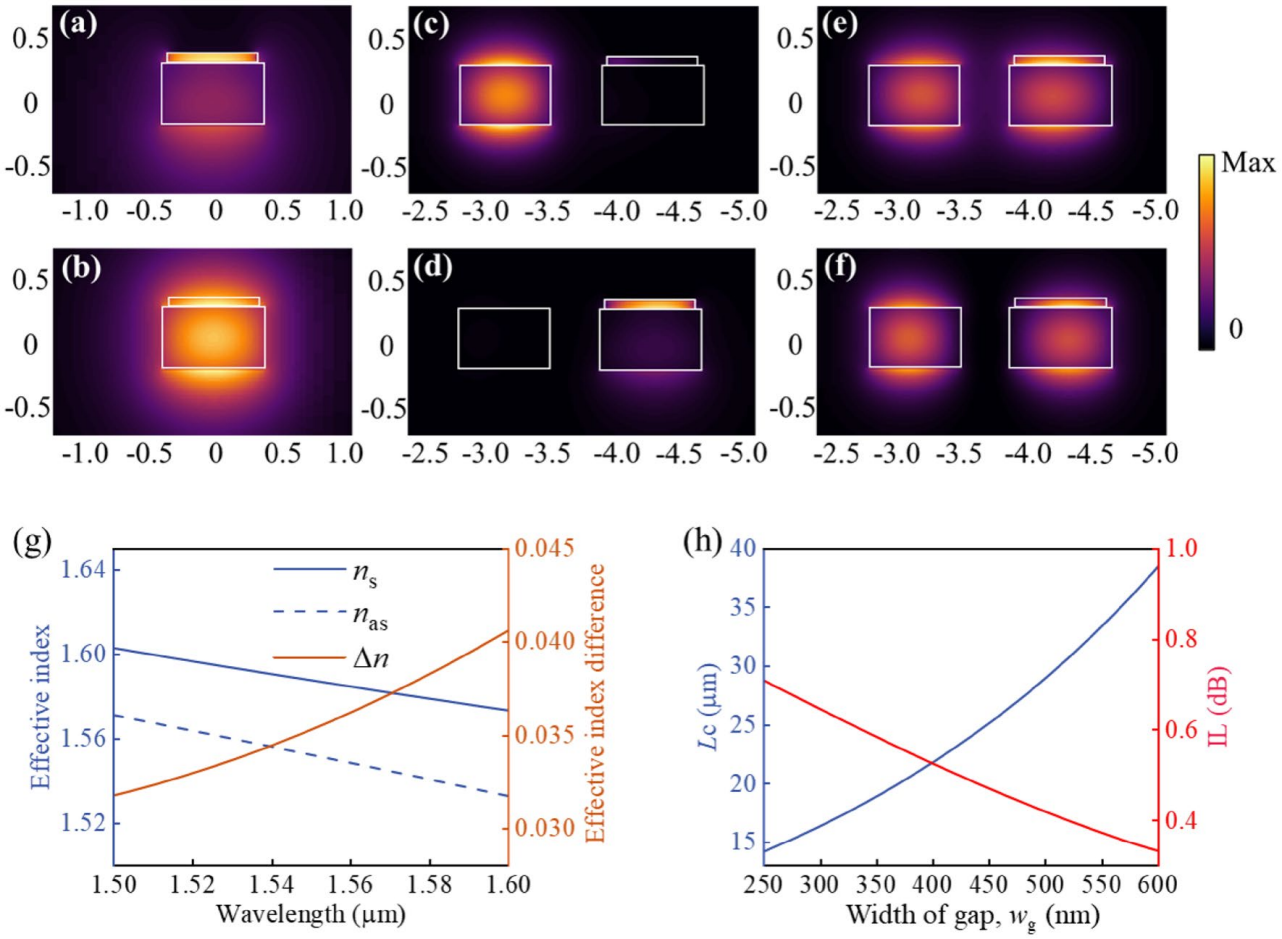
Mode field distribution of a SiN waveguide topped with the TCO layer in the (**a**) pump-off state and (**b**) pump-on state with pump fluence *F* = 0.0569 mJ cm^−2^. The field intensity profile of a two-waveguide configuration with the TCO layer in the pump-off state (**c**,**d**), and in the pump-on state (**e**,**f**). (**g**) Effective index of the symmetric *n*_s_ and antisymmetric *n*_as_ supermodes as functions of wavelength in the pump-on state. (**h**) Coupling length *L*_c_ in the pump-on state and insertion loss IL in the pump-off state as a function of gap width *w*_*g*_.

Figure 5a,b exhibit the modal intensity profile of quasi-transversal magnetic (TM) which can be supported by the single TCO-loaded waveguide in the pump-off state and pump-on state, respectively. Under the pump-off state, the TCO layer with a relatively low index forms a high-index-contrast structure with the SiN, leading to optical field confinement in the TCO layer. While for the pump-on state, the index of TCO is triggered to a relatively high value, allowing the optical field to be distributed mainly around the SiN waveguide. Supermode analyses are performed to give an illustration of the all-optical switching mechanism in the two-waveguide operation system. Figure 5c,d are the supermodes of the two-waveguide system in the pump-off state. Since the TCO layer operates under a low index, the effective index difference between the TCO-loaded waveguide and input waveguide results in phase mismatch, which isolates the two waveguides during light propagation. In contrast, the TCO layer is triggered to a relatively high index under pump excitation, making the effective index of the two waveguides identical and satisfying phase match condition. The phase match therefore will induce strong coupling between the two waveguides with even and odd supermodes (Fig. 5e,f), also referred to as symmetric and antisymmetric modes. Note that the complex effective index of the even and odd supermodes are 1.592 + 0.0016i and 1.556 + 0.0018i, respectively. The simulated effective indices of symmetric mode *n*_s_ and antisymmetric mode *n*_as_ and the difference between (Δ*n*) them as a function of wavelength are plotted in Fig. 5g. The coupling length *L*_c_ is determined by the relationship *L*_c_ = *λ*/(2Δ*n*), which means there is only one suitable coupling length for a given operation wavelength. Thus, the device designed for a specific wavelength cannot meet the coupling requirement perfectly at the other working wavelengths. For the working wavelength of 1.55 μm, the calculated coupling length of the proposed device *L*_c_ = 21.5 μm. Moreover, Fig. 5h indicates the increase of the width of the gap *w*_g_ leads to an increase of *L*_c_ in the pump-on state and a decrease of IL in the pump-off state. Considering the tradeoff between coupling length and IL, the gap width *w*_p_ is selected as 400 nm.

### Transmission analysis

**Figure 6:**
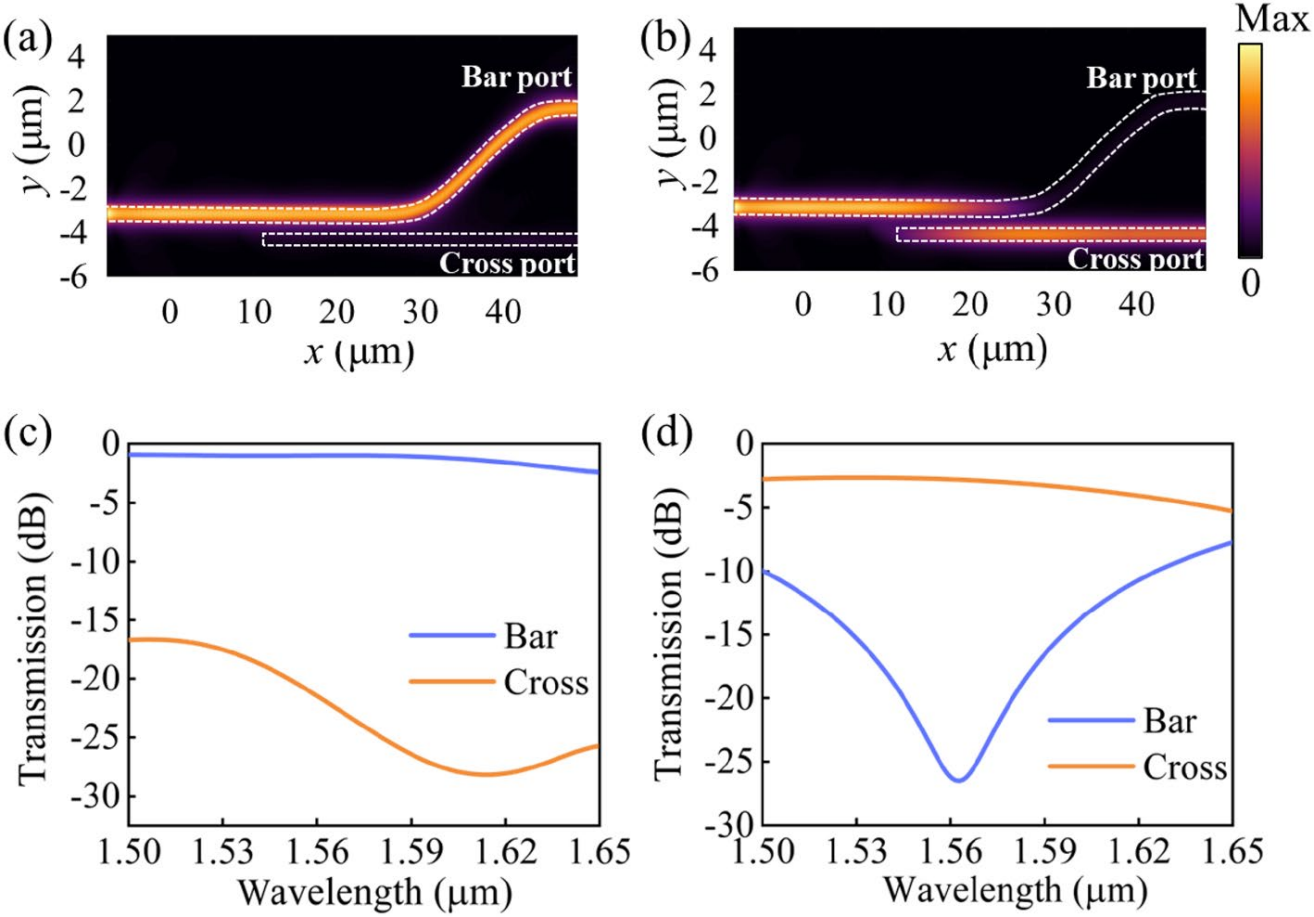
The normalized field intensity distribution along the propagation direction in the pump-off state (**a**) and pump-on state (**b**). Transmission spectra in the Cross port and Bar port in the pump-off state (**c**) and pump-on state (**d**).

The light propagation property is analyzed based on finite-difference time-domain by Lumerical FDTD Solution. The field intensity distribution along the propagation direction of the proposed device in the pump-off state *F* = 0 is depicted in Fig. 6a. The TM-polarized light entirely remains in the input waveguide due to the phase mismatch between the two waveguides. Figure 6c shows the monitored transmission spectra of the Bar port and Cross port under *F* = 0. It is apparent that the optical switch achieves an IL = 1.02 dB and CT = − 18.93 dB at 1.55 μm. For the pump-on state *F* = 0.0569 mJ cm^−2^, the phase match condition in the two-waveguide system is satisfied. As shown in Fig. 6b, it can be seen that the signal light initially propagates only in the input waveguide, and then is evanescently coupled and transferred to the TCO-loaded waveguide. Transmission spectra monitored at the output Cross port and Bar port show that the optical switch attains a low IL = 2.70 dB and a CT = − 19.54 dB at the communication wavelength of 1.55 μm, as shown in Fig. 6d.

**Figure 7:**
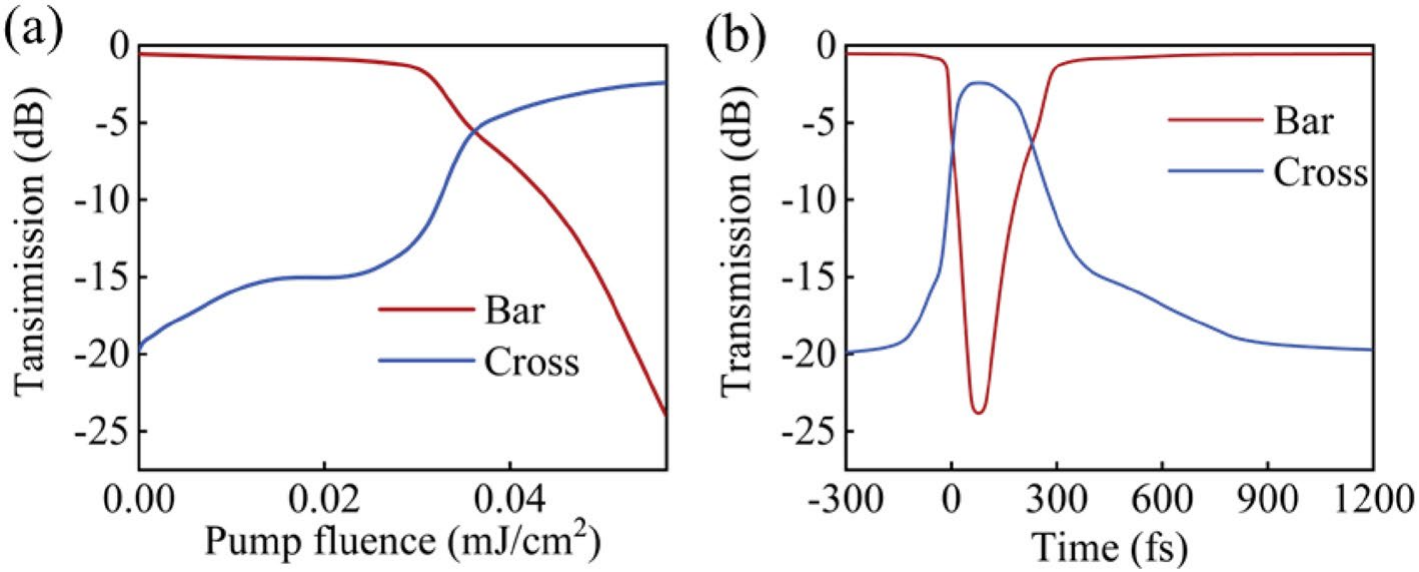
(**a**) Transmission versus pump fluence at the output Bar and Cross port. (**b**) Transient transmission at the output Bar and Cross port.

Figure 7a shows the transmission spectrum of the proposed all-optical switch for various pump fluence *F* at 1.55 μm. One can observe that the increase in pump fluence *F* leads to a decrease in transmission at the Bar port while an increase in transmission at the Cross port. This results from the gradual increase of the TCO index with the increment of *F*, which allows more electrical field to be coupled into the TCO-loaded waveguide. Under pump fluence *F* = 0.0569 mJ cm^−2^, the energy consumption of the switch for per bit is estimated as 14.6 pJ. In practical pump irritation, the illuminated spot will be larger than the designed layer, which leads to an increase in energy consumption per bit. The temporal response of the all-optical switch under *F* = 0.0569 mJ cm^−2^ is illustrated in Fig. 7b. Owing to the ultrafast transient response of the TCO layer, the device features a femtosecond magnitude of switching time. For the Cross port, a rising time of 121 fs and a falling time of 532 fs are obtained, while for the Bar port, a rising time of 163 fs and a falling time of 58 fs are attained. Here, the response time of this switch is strongly consistent with excitation and recovery of hot-electrons in the TCO layer. As extensively reported, ENZ effect utilized here is not limited to TCO layer and generally, it can be achieved by other approaches, for instance, plasmonic metamaterials which can effectively improve energy efficiency and make devices more compact. In summary, the modulation depth for the bar port is 18.93 dB, while that for the cross port is 19.54 dB. The all-optical switching system shows minimal 3-dB broadband over 1527–1594 nm, and features an ultrafast switching time of 221 fs for the bar port and 652 fs for the cross port. To better characterize the performance of the proposed device, we have summarized the recent works and compared the relevant work in recent years shown in Table 1. The proposed all-optical switch based on ADC exhibits the advantages of large modulation depth, wide bandwidth, and ultrafast switching speed. Noticeably, the interface between CdO and silicon nitride causes scattering across grain boundaries and increases the Drude-damping, which leads to an increase of the insertion loss of the proposed device^43,44^. On the other hand, the existence of the phononic interfacial thermal resistance causes the electron–phonon nonequilibrium near the interface between TCO and SiN. The undesired effect reduces the hot electron cooling with a weaker electron–phonon coupling coefficient gep, which may lead to a longer nonlinear response time near the interface^45^.

**Table 1:** Performance comparison with recent works.

Type	Structure	Modulation depth	Bandwidth	Speed	Energy consumption	References
Electro-absorption modulator	Slot waveguide (graphene)	1.6 dB μm^−1^	≥ 6.8 GHz	N.A	418 fJ bit^−1^	^46, 47^
Electro-optical switch	Hyperbolic metamaterial	26 dB	N.A	500 MHz	2.5 aJ bit^−1^	^48^
All-optical modulator	Strip waveguide (hyperbolic metamaterial)	6.33 dB μm^−1^	N.A	9.1 ps	3.7 pJ bit^−1^	^49^
All-optical switch	Metal–insulator–metal nanocavity	120% reflectivity modulation	400 GHz	3 ps	5.2 mJ cm^−2^	^50^
All-optical modulator	2D hybrid perovskites	2% reflectivity modulation	N.A	20 fs	20 pJ μm^−2^	^51^
All-optical modulator	Microring resonator (PtSe_2_)	~ 5 dB	2.4 kHz	284 us	210 mW	^52^
All-optical modulator	Strip waveguide (VO_2_)	1.68 dB μm^−1^	~ 12.5 THz	~ ns	6.4 pJ bit^−1^	^10^
All-optical switch	Au/CdO/HfO_2_/Si capacitor	15.9 dB µm^−1^	N.A	230 fs	13.5 fJ bit^−1^	^53^
Optical switch	Directional coupler (GST)	22 dB (cGST) 10 dB (aGST) coupling length: ~ 30 μm	30 nm	N.A	2 nJ bit^−1^	^39^
Optical switch	Directional coupler (GSST)	50 dB (crystalline state) 27 dB (amorphous state) coupling length: 40 μm	N.A	N.A	N.A	^40^
All-optical switch	Directional coupler (ENZ TCO)	18.93 dB (bar) 19.54 dB (cross) footp coupling length: 21.5 μm	8.25 THz	221 fs (bar) 653 fs (cross)	14.6 pJ bit^−1^	This work

## Conclusion

In conclusion, we have numerically demonstrated an all-optical switch based on ADC using ENZ effect on SiN platform. By calculation, in the ENZ region, this intensity-dependent refractive index and ultrafast transient response of the TCO layer under different pump fluence are assessed. The mode and 3D transmission  analyses are used to investigated the performance of the device. The results show that, under an excitation pump fluence of 0.0569 mJ cm^−2^ at telecom wavelength, the switch features low insertion loss, low crosstalk, broadband, and ultrafast transition time both in the pump-off and pump-on state near ENZ region. The results of this work demonstrate for the first time the application of the ENZ effect in integrated waveguide switch, offering a possibility for the design of switching components in high-speed all-optical signal processing chips.

## Methods

### Non-parabolic conduction of TCO

The plasma frequency *ω*_p_ can be written as^27^:

2 $$\omega_{{\text{p}}} (T_{e} )^{2} = \frac{{e^{2} }}{{3m\pi^{2} }}\int_{0}^{\infty } {dE\left( {\frac{2m}{{\hbar^{2} }}(E + CE^{2} )} \right)^{\frac{3}{2}} (1 + 2CE)^{ - 1} \left( { - \frac{{\partial f_{0} (E,T_{{e{\kern 1pt} }} )}}{\partial E}} \right)} ,$$

where *m* = 0.12 *m*_0_ (*m*_0_ the free electron mass) denotes the effective mass, *ħ* is the reduced Plank’s constant, *C* = 0.34 eV^−1^^19^ determines the structure of non-parabolic conduction, and the function *f*_0_(*E*, *T*_*e*_) is the Fermi–Dirac distribution function determined by electron temperature *T*_*e*_ and the electron energy *E*. Here, electron-temperature-dependence of *f*_0_ is mainly due to the fact that *T*_*e*_ can produce a change in electro-chemical potential and thereby modifying Fermi–Dirac function. Note that, the modification of the Fermi–Dirac distribution induced by *T*_*e*_ is more markable in degenerately doped semiconductors than in noble metals. The reasons originate from the lower heat capacity of electrons in CdO as well as its non-parabolic conduction structure. Therefore, the phenomenon gives rise to an electron-temperature-dependent plasma frequency *ω*_p_(*T*_*e*_).

### Two-temperature-model

To calculate the transient response of the TCO layer, the following two-temperature model is used^11,27^:

3 $$\begin{aligned} C_{e} (T_{e} )\frac{{\partial T_{e} (t)}}{\partial t} = & - g_{ep} (T_{e} (t) - T_{l} (t)) + \frac{N(t)}{{\tau_{ee} (t)}}, \\ C_{l} \frac{{\partial T_{l} (t)}}{\partial t} = & g_{ep} (T_{e} (t) - T_{l} (t)) + \frac{N(t)}{{\tau_{{e{\text{p}}}} (t)}}, \\ \frac{\partial N(t)}{{\partial t}} = & - \frac{N(t)}{{\tau_{ee} (t)}} - \frac{N(t)}{{\tau_{ep} (t)}} + P(t), \\ \end{aligned}$$

where *T*_*l*_ is the lattice temperature, *τ*_*ee*_ (*τ*_*e*p_) is the electron–electron (electron–phonon) relaxation time, and *C*_*e*_ (*C*_*l*_) is the heat capacity of free electron (lattice). *N* is the non-thermal energy density stored in the excited electrons, and *g*_*ep*_ denotes the electron–phonon coupling coefficient. Here, *P* is time-dependent absorbed energy in the material, and *C*_*e*_, *g*_*ep*_, *P* can be expressed as follow respectively^11^:

4 $$C_{e} = \frac{{3\pi^{2} n_{e} k_{{\text{B}}} T_{e} }}{{\sqrt {36T_{{\text{F}}}^{2} + 4\pi^{4} T_{e}^{2} } }},$$

5 $$g_{ep} { = }0.562n_{e} \frac{{k_{{\text{B}}}^{2} \Theta_{{\text{D}}}^{2} \nu_{{\text{F}}} }}{{L_{{\text{f}}} T_{l} E_{{\text{F}}} }},$$

6 $$P(t) = \frac{F}{{\tau_{{\text{p}}} }}\alpha e^{{ - 2\left( {\frac{t}{{\tau_{{\text{p}}} }}} \right)^{2} }} .$$

In Eq. (4), *k*_B_ is the Boltzmann constant; *n*_*e*_ = 6.1 × 10^20^ cm^-3^ is the total number electrons in the conduction band and maintained as a constant under a proper thermal excitation; and *T*_F_ is the Fermi temperature obtained from Ref.^27^. In Eq. (5), Θ_D_ is the Debye temperature^54^, *v*_F_ is the Fermi velocity^55^, *E*_F_ is the Fermi level and *L*_f_ is the electron mean free path^56^. In Eq. (6), *F* is the pump fluence, *τ*_p_ is the pump pulse duration set as 100 fs and *α* is the attenuation coefficient of CdO estimated from Ref.^57^. In the calculation of TTM, consider that the lattice has a much higher heat capacity *C*_*l*_ of than the heat capacity of electron *C*_*e*_, we can take it as a constant with *C*_*l*_ = 2.9 × 10^6^ J·m^−3^ K^−1^^58^.

## Data Availability

The data that support the findings of this study are available from the corresponding author upon resonable request.
